# Supplementary material for: The effect of perceived interracial competition on psychological outcomes
Source: PLoS One. 2021 Jan 29;16(1):e0245671. doi: 10.1371/journal.pone.0245671 (PMC7845962; doi:10.1371/journal.pone.0245671)
Supplement: S1 Appendix — (DOCX) [file pone.0245671.s001.docx]

**Appendix S1.**

Perceived Racial Competition

Think about **the ZIP code you live in**. Please indicate how much you agree with each of the following statements.

1. In my ZIP code, Blacks and Whites seem to value competition with each other.
2. In my ZIP code, it seems that Blacks and Whites are competing with each other.
3. In my ZIP code, Blacks and Whites seem to share the feeling that competing with each other is important.
4. In my ZIP code, it seems that Blacks are competing with Whites and Whites are competing with Blacks.
5. In my ZIP code, I feel that Blacks and Whites are being compared with one another.

Seven-point scale (1 = “Not at all”; 4 = “Somewhat”; 7 = “Completely”)

Perceived Discrimination

**In your ZIP code,** how often do the following things happen to people **because of their race?**

1. Being treated with less courtesy than others.
2. Being treated with less respect than others.
3. Receiving poorer service than others in restaurants or stores.
4. People acting as if he/she is not smart.
5. People acting as if they are afraid of him/her.
6. Others feeling they are better than him/her.
7. Others thinking that he/she is dishonest.
8. Being called names or insulted.
9. Being threatened or harassed.

Seven-point scale (1 = “Never”; 4 = “Sometimes”; 7 = “Frequently”)

Perceived Behavioral Avoidance

Rate how much you agree with each statement on a scale from 1 (strongly disagree) to 7 (strongly agree).

1. In my ZIP code, Black and White people avoid having conversations with each other.
2. In my ZIP code, Black and White people avoid having friendships with each other.
3. In my ZIP code, Black and White people avoid spending leisure time with each other.
4. In my ZIP code, Black and White people avoid having romantic relationships with each other.
5. In my ZIP code, Black and White people avoid having each other as neighbors.
6. In my ZIP code, Black and White people avoid shopping in stores with each other.
7. In my ZIP code, Black and White people avoid attending events with each other.
8. In my ZIP code, Black and White people avoid working with each other.
9. In my ZIP code, if Black and White people had to interact with each other, they would end the interaction as soon as possible.
10. In my ZIP code, if Black and White people had a choice, they would rather not interact with each other.
11. In my ZIP code, if Black and White people can avoid interacting with each other, they do.

Perceived Intergroup Anxiety

**Intergroup Anxiety**

1. In my ZIP code, Black and White people feel nervous about interacting with each other.
2. In my ZIP code, Black and White people seem to feel uneasy about interacting with each other.
3. In my ZIP code, Black and White people feel tense about interacting with each other.
4. In my ZIP code, Black and White people feel bothered about interacting with each other.

Items are rated on a scale from 1 (Strongly disagree), 4 (Somewhat), 7 (Strongly agree).

Perceived Interracial Trust

1. In my ZIP code, Black and White people are basically honest with each other.
2. In my ZIP code, Black and White people view each other as trustworthy.
3. In my ZIP code, Black and White people view each other as basically good and kind.
4. In my ZIP code, Black and White people are trustful of each other.

Respondents were asked to give a score ranging from 1 to 7, where 1 represented very low trust in others and 7 very high trust.

Seven-point scale (1 = “Not at all”; 4 = “Somewhat”; 7 = “Completely”)

Perceived Racial Income Inequality

Think about **the town/city you live in**. Please indicate how much you agree with each of the following statements.

1. In my town/city, there is a huge gap between Blacks and Whites.
2. In my town/city, those in the top 1% of income earners are more likely to be White than Black.
3. In my town/city, the income disparity between Blacks and Whites is large.

Seven-point scale (1 = “Not at all”; 4 = “Somewhat”; 7 = “Completely”)

**Social Dominance Orientation (SDO)**

Show how much you favor or oppose each idea below by selecting a number from 1 to 7 on the scale below. You can work quickly; your first feeling is generally best. (1=Strongly Oppose; 7 = Strongly Favor)

*Pro-trait dominance:*

1. An ideal society requires some groups to be on top and others to be on the bottom.
2. Some groups of people are simply inferior to other groups.

*Con-trait dominance:*

1. No one group should dominate in society.
2. Groups at the bottom are just as deserving as groups at the top.

*Pro-trait antiegalitarianism:*

1. Group equality should not be our primary goal.
2. It is unjust to try to make groups equal.

*Con-trait antiegalitarianism:*

1. We should do what we can to equalize conditions for different groups.
2. We should work to give all groups an equal chance to succeed.

**Support for Economic Inequality Scale (SEI)**

Please indicate how much you agree with each of the following statements.

1. The negative consequences of economic inequality have been largely exaggerated.
2. Economic inequality is causing many of the world’s problems. (R)
3. I am very disturbed by the amount of economic inequality in the world today. (R)
4. Economic inequality is not a problem.
5. We need to do everything possible to reduce economic inequality in the world today. (R)

1 = Strongly Disagree to 7 = Strongly Agree

**Economic system justification (ESJ)**

Please select a number to indicate the degree to which you agree or disagree with each of the following statements:

1. If people work hard, they almost always get what they want.
2. The existence of widespread economic differences does not mean that they are inevitable. (re- verse-scored)
3. Laws of nature are responsible for differences in wealth in society.
4. There are many reasons to think that the economic system is unfair. (reverse-scored)
5. It is virtually impossible to eliminate poverty.
6. Poor people are not essentially different from rich people (reverse-scored)
7. Most people who don’t get ahead in our society should not blame the system; they have only themselves to blame
8. Equal distribution of resources is a possibility for our society (reverse-scored);
9. Social class differences reflect differences in the natural order of things;
10. Economic differences in the society reflect an illegitimate distribution of resources (reverse-scored);
11. There will always be poor people, because there will never be enough jobs for everybody;
12. Economic positions are legitimate reflections of people’s achievements;
13. If people wanted to change the economic system to make things equal, they could (reverse-scored);
14. Equal distribution of resources is unnatural;
15. It is unfair to have an economic system which produces extreme wealth and extreme poverty at the same time (reverse-scored);
16. There is no point in trying to make incomes more equal; and
17. There are no inherent differences between rich and poor; it is purely a matter of the circumstances into which you are born (reverse-scored).

1 = Strongly Disagree to 9 = Strongly Agree

**Group Identification**

Please indicate how much you agree with each of the following statements.

1. I identify with other [Black/White] people.
2. I see myself as a [Black/White] person.
3. I am glad to be a [Black/White] person.
4. I feel strong ties with [Black/White] people.

1 = Strongly Disagree to 7 = Strongly Agree

**Trait Competitiveness**

Please indicate the extent to which you agree or disagree with each of the following statements.

1 = Disagree Strongly, 5 = Agree Strongly

1. I feel that winning is important in both work and games.
2. I enjoy working in situations involving competition with others.
3. It is important for me to perform better than others on a task.
4. I try harder when I'm in competition with other people.
5. It annoys me when other people perform better than I do.
