# Supplementary material for: The effect of perceived interracial competition on psychological outcomes
Source: PLoS One. 2021 Jan 29;16(1):e0245671. doi: 10.1371/journal.pone.0245671 (PMC7845962; doi:10.1371/journal.pone.0245671)
Supplement: S1 File — (DOCX) [file pone.0245671.s002.docx]

## **Footnotes**

^1^ Non-White and non-Black participants were not excluded from Study 1 because when analyses were conducted, these participants were erroneously not filtered. To maintain transparency and statistical power, we kept these participants, as filtering participants to include only White and Black participants reduces the sample size to N = 697. When examining only White and Black participants, effects of condition on each outcome remain significant and consistent (*ps <* .004).

^2^ As seen in the preregistration, additional moderators were examined to explore potential effects. We placed the results for these potential moderators in Supplementary Materials given space considerations (see Tables S3-S9). We encourage others to pursue the role of individual differences on perceptions of [interracial] competition, and hope that these supplementary results help spark this interest.

# **Study 1**

### **Moderators**

**Racial income gap (RIGap).** Because participants enter their ZIP-code in order to receive the normative feedback induction, we were able to use their ZIP-codes to calculate the degree of racial income inequality in their area. The RIGap was calculated using the 2016 American Community Survey’s five-year estimates (the most recent estimates available during data collection). These data are publicly available from the U.S. Census Bureau (see https://data.census.gov/cedsci/). A gap score was calculated using the income difference between Blacks and Whites in a given ZIP-code area. Higher values correspond to Whites having more income than Blacks.

**Exploratory Analyses – Racial income gap and racial income gap x condition interaction.**

Given the hierarchical structure of the data (participants nested in their ZIP-codes), we first built a multilevel model having no predictor, using each of the outcomes separately. We first calculated the *design effect* (*DEFF*; [1]); this assessed the impact of ZIP-code clustering on estimation of the standard error. A *DEFF* > 2 indicates that the impact of ZIP-code clustering is substantial and that multilevel analyses should be preferred over single-level analyses [2]. Across each of the outcomes, the design effects ranged from 1.01-1.08. As the design effect was well below threshold, the incidence of ZIP-code clustering was negligible, indicating that single-level and multilevel analyses were expected to produce comparable results. Thus, we used standard single-level analyses.

See Table S1 and S2 for results. Hierarchical multiple regression was used to examine the association between ZIP-code level racial income gap and the outcome variables, controlling for condition. RIGap predicted perceptions of racial competition, *β* = .08, [.01, .14], *p* = .025, behavioral avoidance, *β* = .07, [.004, .14], *p* = .039, and intergroup anxiety, *β* = .07, [.004, .14], *p* = .037. The RIGap x condition interaction was not significant for any of the outcomes.

# **Study 2**

### **Moderators**

**Social Dominance Orientation (SDO).** The eight-item version of the Social Dominance Scale [3] was used (e.g. “*Some groups of people are simply inferior to other group*,”; 1 = *Strongly Oppose,* 7 = *Strongly Favor; α* = .85).

**Support for Economic Inequality.** The five-item Support for Economic Inequality Scale [4] was used to measure the degree to which individuals support income inequality (e.g. “*The negative consequences of economic inequality have been largely exaggerated*”; 1 = *Strongly Disagree*, 7 = *Strongly Agree*; *α* = .84).

**Economic System Justification.** The seventeen-item Economic System Justification scale [5] was used to measure the ideological tendency to legitimize economic inequality (e.g. “*If people work hard, they almost always get what they want*”; 1 = *Strongly disagree*, 9 = *Strongly agree*; *α* = .77).

**Ingroup Identification.** The four-item group identification measure [6] was used to assess ingroup identification and membership (e.g. “*I identify with other Black/White people*”; 1 = *strongly disagree*, 7 = *strongly agree*; *α* = .88). For this scale, participants read items specific to their own self-identified race based on demographic responses screened *a priori*.

**Trait Competitiveness.** The five-item Competitiveness Scale [7] was used as a measure of trait competitiveness (e.g. “*I enjoy working in situations involving competition with others*”; 1 = *Disagree Strongly*, 5 = *Agree Strongly*; *α* = .84).

### **Moderators of Condition and Race Effects**

Hierarchical multiple regression analyses were used to examine the effects of condition and race on the outcome variables with each moderator separately. Condition, race, and each moderator variable were entered in step 1, followed by condition x moderator and race x moderator interaction terms in step 2. See Tables S3-S7 for results.

**Social Dominance Orientation (SDO).** Condition x SDO predicted perceptions of interracial competition, *β* = -.06, [-.11, .02], *p* = .004, discrimination, *β* = -.07, [-.12, -.02], *p* = .003, and mistrust, *β* = -.06, [-.10, -.01], *p* = .019. The effects of condition on perceived interracial competition and discrimination were weaker for those higher in SDO. For perceptions of interracial mistrust, the condition main effect was only significant for those lower in SDO.

**Support for Economic Inequality (SEI).** Condition x SEI predicted perceptions of interracial competition, *β* = -.07, [-.12, -.03], *p* = .001, discrimination, *β* = -.08, [-.12, -.03], *p* = .001, and, marginally, perceptions of behavioral avoidance, *β* = -.04, [-.09, .01], *p* = .086, intergroup anxiety, *β* = -.04, [-.09, .005], *p* = .079, and interracial mistrust, *β* = -.04, [-.09, .002], *p* = .058. The effects of condition on these outcomes were weaker for those higher in SEI.

**Economic System Justification (ESJ).** Condition x ESJ predicted perceptions of interracial competition, *β* = -.04, [-.09, .00], *p* = .048, and discrimination, *β* = -.07, [-.12, -.03], *p* = .002. The effects of condition on perceived interracial competition and discrimination were weaker for those higher in ESJ.

**Ingroup Identification (INGROUP).** No condition x INGROUP interaction effects reached significance.

**Trait Competitiveness.** Condition x trait competitiveness predicted perceptions of discrimination, *β* = -.06, [-.10, -.01], *p* = .015, interracial mistrust, *β* = -.05, [-.096, -.004], *p* = .035, and, marginally, perceptions of behavioral avoidance, *β* = -.05, [-.09, .001], *p* = .054. The effects of condition on perceived discrimination and behavioral avoidance were weaker for those higher in trait competitiveness, while the interaction effect on interracial mistrust revealed that the condition effect was only significant for those lower in trait competitiveness.

### **Main effect analyses – Individual Difference Moderators on negative psychological outcomes.**

For main effects of the moderating variables on negative psychological outcomes, see Tables S3-S7. For Descriptive statistics and intercorrelations for perceived interracial competition, the race-based psychological outcomes, and all moderator variables examined, see Table S8. Lastly, for simple slopes for the condition x moderator interactions on race-based psychological outcomes, see Table S9.

# **References**

1. Kish L. Survey sampling. 1965.

2. Muthen BO, Satorra A. Complex Sample Data in Structural Equation Modeling. Sociol Methodol. 1995;25: 267. doi:10.2307/271070

3. Ho AK, Sidanius J, Kteily N, Sheehy-Skeffington J, Pratto F, Henkel KE, et al. The Nature of Social Dominance Orientation: Theorizing and Measuring Preferences for Intergroup Inequality Using the New SDO7 Scale. J Pers Soc Psychol. 2015;109: 1003–1028. doi:10.1037/pspi0000033

4. Wiwad D, Mercier B, Maraun MD, Robinson AR, Piff PK, Aknin LB, et al. The support for economic inequality scale: Development and adjudication. PLoS One. 2019;14: e0218685. doi:10.1371/journal.pone.0218685

5. Jost JT, Thompson EP. Group-based dominance and opposition to equality as independent predictors of self-esteem, ethnocentrism, and social policy attitudes among african americans and european americans. J Exp Soc Psychol. 2000;36: 209–232. doi:10.1006/jesp.1999.1403

6. Doosje B, Ellemers N, Spears R. Perceived intragroup variability as a function of group status and identification. J Exp Soc Psychol. 1995;31: 410–436. doi:10.1006/jesp.1995.1018

7. Helmreich RL, Spence JT. The work and family orientation questionnaire. An Object instruments to assess components Achiev Motiv attitudes Towar Fam carreer JSAS Cat Sel Doc Psychol. 1978;8: 35.

Table S1

*Study 1: Descriptive statistics and intercorrelations for racial income gap, perceived racial competition, and the race-based psychological outcomes*

|  | Descriptive statistics | | | | Pairwise intercorrelations | | | | | |
| --- | --- | --- | --- | --- | --- | --- | --- | --- | --- | --- |
|  | α | *M* | *SD* | 1 | | 2 | 3 | 4 | 5 | 6 |
| 1. Racial income Gap | – | $11,734 | $16,218 | – | |  |  |  |  |  |
| 1. Perceived racial competition | .95 | 3.07 | 1.73 | .08* | | – |  |  |  |  |
| 1. Perceived discrimination | .97 | 3.52 | 1.57 | .05 | | .55*** | – |  |  |  |
| 1. Perceived behavioral avoidance | .97 | 2.87 | 1.65 | .07* | | .63*** | .71*** | – |  |  |
| 1. Perceived intergroup anxiety | .97 | 3.10 | 1.78 | .07* | | .63*** | .75*** | .86*** | – |  |
| 1. Perceived interracial mistrust | .95 | 3.36 | 1.48 | .04 | | .08* | .35*** | .26*** | .35*** | – |

*Notes*: ****p* < .001, ***p* < .01, **p* < .05.

Table S2

*Study 1: Standardized coefficient estimates of condition on race-based outcomes moderated by RIGap.*

|  | PCOMP | | | | DISCRIM | | | | AVOID | | | | ANX | | | | MISTRUST | | | |
| --- | --- | --- | --- | --- | --- | --- | --- | --- | --- | --- | --- | --- | --- | --- | --- | --- | --- | --- | --- | --- |
|  | Step 1 | | Step 2 | | Step 1 | | Step 2 | | Step 1 | | Step 2 | | Step 1 | | Step 2 | | Step 1 | | Step 2 | |
| Variable | *β* | *SE* | *β* | *SE* | *β* | *SE* | *β* | *SE* | *β* | *SE* | *β* | *SE* | *β* | *SE* | *β* | *SE* | *β* | *SE* | *β* | *SE* |
| Condition | .30^***^ | .03 | .30^***^ | .03 | .18^***^ | .03 | .18^***^ | .03 | .15^***^ | .03 | .15^***^ | .03 | .18^***^ | .03 | .18^***^ | .03 | .11^**^ | .04 | .11^**^ | .04 |
| RIGap | .08^*^ | .03 | .08^*^ | .03 | .05 | .03 | .05 | .04 | .07^*^ | .03 | .08^*^ | .04 | .07^*^ | .03 | .08^*^ | .04 | .04 | .04 | .05 | .04 |
| Condition x RIGap |  |  | -.02 | .04 |  |  | -.01 | .04 |  |  | -.01 | .04 |  |  | -.02 | .04 |  |  | -.06 | .04 |

*Notes*: ****p* < .001, ***p* < .01, **p* < .05; RIGap = Racial income gap

Table S3

*Study 2: Standardized coefficient estimates of condition and race on race-based outcomes moderated by social dominance orientation.*

|  | PCOMP | | | | DISCRIM | | | | AVOID | | | | ANX | | | | MISTRUST | | | |
| --- | --- | --- | --- | --- | --- | --- | --- | --- | --- | --- | --- | --- | --- | --- | --- | --- | --- | --- | --- | --- |
|  | Step 1 | | Step 2 | | Step 1 | | Step 2 | | Step 1 | | Step 2 | | Step 1 | | Step 2 | | Step 1 | | Step 2 | |
| Variable | *β* | *SE* | *β* | *SE* | *β* | *SE* | *β* | *SE* | *β* | *SE* | *β* | *SE* | *β* | *SE* | *β* | *SE* | *β* | *SE* | *β* | *SE* |
| Condition | .35^***^ | .02 | .35^***^ | .02 | .20^***^ | .02 | .20^***^ | .02 | .15^***^ | .02 | .15^***^ | .02 | .20^***^ | .02 | .20^***^ | .02 | .11^***^ | .02 | .11^***^ | .02 |
| Race | -.15^***^ | .02 | -.15^***^ | .02 | -.20^***^ | .02 | -.20^***^ | .02 | -.10^***^ | .02 | -.10^***^ | .02 | -.11^***^ | .02 | -.11^***^ | .02 | -.26^***^ | .02 | -.26^***^ | .02 |
| SDO | .17^***^ | .02 | .17^***^ | .02 | .04† | .02 | .04 | .02 | .28^***^ | .02 | .27^***^ | .02 | .17^***^ | .02 | .18^***^ | .03 | -.02 | .02 | -.04 | .02 |
| Condition x SDO |  |  | -.07^**^ | .02 |  |  | -.07^**^ | .02 |  |  | -.04† | .02 |  |  | -.03 | .02 |  |  | -.05^*^ | .02 |
| Race x SDO |  |  | .02 | .02 |  |  | .02 | .02 |  |  | .03 | .02 |  |  | -.01 | .02 |  |  | .06^**^ | .02 |

*Notes*: ****p* < .001, ***p* < .01, **p* < .05, †*p* < .10

Table S4

*Study 2: Standardized coefficient estimates of condition and race on race-based outcomes moderated by support for economic inequality.*

|  | PCOMP | | | | DISCRIM | | | | AVOID | | | | ANX | | | | MISTRUST | | | |
| --- | --- | --- | --- | --- | --- | --- | --- | --- | --- | --- | --- | --- | --- | --- | --- | --- | --- | --- | --- | --- |
|  | Step 1 | | Step 2 | | Step 1 | | Step 2 | | Step 1 | | Step 2 | | Step 1 | | Step 2 | | Step 1 | | Step 2 | |
| Variable | *β* | *SE* | *β* | *SE* | *β* | *SE* | *β* | *SE* | *β* | *SE* | *β* | *SE* | *β* | *SE* | *β* | *SE* | *β* | *SE* | *β* | *SE* |
| Condition | .35^***^ | .02 | .35^***^ | .02 | .20^***^ | .02 | .20^***^ | .02 | .15^***^ | .02 | .15^***^ | .02 | .20^***^ | .02 | .20^***^ | .02 | .11^***^ | .02 | .11^***^ | .02 |
| Race | -.14^***^ | .02 | -.14^***^ | .02 | -.16^***^ | .02 | -.16^***^ | .02 | -.08^**^ | .02 | -.08^**^ | .02 | -.09^***^ | .02 | -.09^***^ | .02 | -.25^***^ | .02 | -.24^***^ | .02 |
| SEI | .08^***^ | .02 | .08^***^ | .02 | -.10^***^ | .02 | -.11^***^ | .03 | .12^***^ | .02 | .12^***^ | .03 | .05† | .02 | .05^*^ | .03 | -.06^*^ | .02 | -.08^**^ | .03 |
| Condition x SEI |  |  | -.07^**^ | .02 |  |  | -.08^**^ | .02 |  |  | -.04† | .02 |  |  | -.04† | .02 |  |  | -.04† | .02 |
| Race x SEI |  |  | .02 | .02 |  |  | .04† | .02 |  |  | .00 | .03 |  |  | -.01 | .02 |  |  | .08^***^ | .02 |

*Notes*: ****p* < .001, ***p* < .01, **p* < .05, †*p* < .10

Table S5

*Study 2: Standardized coefficient estimates of condition and race on race-based outcomes moderated by economic system justification.*

|  | PCOMP | | | | DISCRIM | | | | AVOID | | | | ANX | | | | MISTRUST | | | |
| --- | --- | --- | --- | --- | --- | --- | --- | --- | --- | --- | --- | --- | --- | --- | --- | --- | --- | --- | --- | --- |
|  | Step 1 | | Step 2 | | Step 1 | | Step 2 | | Step 1 | | Step 2 | | Step 1 | | Step 2 | | Step 1 | | Step 2 | |
| Variable | *β* | *SE* | *β* | *SE* | *β* | *SE* | *β* | *SE* | *β* | *SE* | *β* | *SE* | *β* | *SE* | *β* | *SE* | *β* | *SE* | *β* | *SE* |
| Condition | .35^***^ | .02 | .35^***^ | .02 | .20^***^ | .02 | .20^***^ | .02 | .15^***^ | .02 | .15^***^ | .02 | .20^***^ | .02 | .20^***^ | .02 | .11^***^ | .02 | .11^***^ | .02 |
| Race | -.14^***^ | .02 | -.14^***^ | .02 | -.18^***^ | .02 | -.18^***^ | .02 | -.07^**^ | .02 | -.07^**^ | .02 | -.09^***^ | .02 | -.09^***^ | .02 | -.25^***^ | .02 | -.24^***^ | .02 |
| ESJ | .19^***^ | .02 | .19^***^ | .02 | -.05^*^ | .02 | -.06^*^ | .02 | .18^***^ | .02 | .17^***^ | .02 | .11^***^ | .02 | .11^***^ | .02 | -.08^**^ | .02 | -.09^***^ | .02 |
| Condition x ESJ |  |  | -.05^*^ | .02 |  |  | -.07^**^ | .02 |  |  | -.03 | .02 |  |  | -.02 | .02 |  |  | -.03 | .02 |
| Race x ESJ |  |  | .01 | .02 |  |  | .04 | .02 |  |  | .03 | .02 |  |  | .01 | .02 |  |  | .05^*^ | .02 |

*Notes*: ****p* < .001, ***p* < .01, **p* < .05, †*p* < .10

Table S6

*Study 2: Standardized coefficient estimates of condition and race on race-based outcomes moderated by ingroup identification.*

|  | PCOMP | | | | DISCRIM | | | | AVOID | | | | ANX | | | | MISTRUST | | | |
| --- | --- | --- | --- | --- | --- | --- | --- | --- | --- | --- | --- | --- | --- | --- | --- | --- | --- | --- | --- | --- |
|  | Step 1 | | Step 2 | | Step 1 | | Step 2 | | Step 1 | | Step 2 | | Step 1 | | Step 2 | | Step 1 | | Step 2 | |
| Variable | *β* | *SE* | *β* | *SE* | *β* | *SE* | *β* | *SE* | *β* | *SE* | *β* | *SE* | *β* | *SE* | *β* | *SE* | *β* | *SE* | *β* | *SE* |
| Condition | .35^***^ | .02 | .35^***^ | .02 | .20^***^ | .02 | .20^***^ | .02 | .15^***^ | .02 | .15^***^ | .02 | .20^***^ | .02 | .20^***^ | .02 | .11^***^ | .02 | .11^***^ | .02 |
| Race | -.08^***^ | .02 | -.09^***^ | .02 | -.18^***^ | .03 | -.17^***^ | .03 | -.04 | .03 | -.04 | .03 | -.07^**^ | .03 | -.07^**^ | .03 | -.28^***^ | .03 | -.27^***^ | .03 |
| INGROUP | .10^***^ | .02 | .09^***^ | .03 | .03 | .03 | .05† | .03 | .04 | .03 | .03 | .03 | .03 | .03 | .03 | .03 | -.04† | .03 | -.03 | .03 |
| Condition x INGROUP |  |  | .01 | .02 |  |  | .02 | .02 |  |  | .00 | .02 |  |  | .02 | .02 |  |  | .02 | .02 |
| Race x INGROUP |  |  | .02 | .02 |  |  | -.05† | .02 |  |  | .03 | .03 |  |  | .02 | .03 |  |  | -.04 | .02 |

*Notes*: ****p* < .001, ***p* < .01, **p* < .05, †*p* < .10

Table S7

*Study 2: Standardized coefficient estimates of condition and race on race-based outcomes moderated by trait competitiveness.*

|  | PCOMP | | | | DISCRIM | | | | AVOID | | | | ANX | | | | MISTRUST | | | |
| --- | --- | --- | --- | --- | --- | --- | --- | --- | --- | --- | --- | --- | --- | --- | --- | --- | --- | --- | --- | --- |
|  | Step 1 | | Step 2 | | Step 1 | | Step 2 | | Step 1 | | Step 2 | | Step 1 | | Step 2 | | Step 1 | | Step 2 | |
| Variable | *β* | *SE* | *β* | *SE* | *β* | *SE* | *β* | *SE* | *β* | *SE* | *β* | *SE* | *β* | *SE* | *β* | *SE* | *β* | *SE* | *β* | *SE* |
| Condition | .35^***^ | .02 | .35^***^ | .02 | .20^***^ | .02 | .20^***^ | .02 | .15^***^ | .02 | .15^***^ | .02 | .20^***^ | .02 | .20^***^ | .02 | .11^***^ | .02 | .11^***^ | .02 |
| Race | -.11^***^ | .02 | -.11^***^ | .02 | -.18^***^ | .02 | -.18^***^ | .02 | -.04† | .02 | -.04† | .02 | -.07^**^ | .02 | -.07^**^ | .02 | -.27^***^ | .02 | -.27^***^ | .02 |
| TRAITCOMP | .20^***^ | .02 | .20^***^ | .02 | .14^***^ | .02 | .14^***^ | .02 | .12^***^ | .02 | .12^***^ | .02 | .13^***^ | .02 | .13^***^ | .02 | -.08^**^ | .02 | -.08^***^ | .02 |
| Condition x TCOMP |  |  | -.03 | .02 |  |  | -.06^*^ | .02 |  |  | -.05† | .02 |  |  | -.03 | .02 |  |  | -.05^*^ | .02 |
| Race x TCOMP |  |  | .01 | .02 |  |  | -.01 | .02 |  |  | .02 | .02 |  |  | .02 | .02 |  |  | -.01 | .02 |

*Notes*: ****p* < .001, ***p* < .01, **p* < .05, †*p* < .10

Table S8

*Study 2: Descriptive statistics and intercorrelations for perceived interracial competition, the race-based psychological outcomes, and moderator variables*

|  | Descriptive statistics | | | | Pairwise intercorrelations | | | | |  |  |  |  |
| --- | --- | --- | --- | --- | --- | --- | --- | --- | --- | --- | --- | --- | --- |
|  | α | *M* | *SD* | 1 | 2 | 3 | 4 | 5 | 6 | 7 | 8 | 9 | 10 |
| 1. Racial income Gap | – | $10,340 | $14,902 | – |  |  |  |  |  |  |  |  |  |
| 2. Perceived interracial competition | .95 | 3.06 | 1.68 | .04 | – |  |  |  |  |  |  |  |  |
| 3. Perceived discrimination | .96 | 3.52 | 1.53 | .03 | .53*** | – |  |  |  |  |  |  |  |
| 4. Perceived behavioral avoidance | .97 | 2.65 | 1.50 | .04 | .55*** | .63*** | – |  |  |  |  |  |  |
| 5. Perceived intergroup anxiety | .97 | 2.93 | 1.66 | .05† | .55*** | .68*** | .82*** | – |  |  |  |  |  |
| 6. Perceived interracial mistrust | .95 | 3.67 | 1.48 | -.04 | .17*** | .42*** | .29*** | .38*** | – |  |  |  |  |
| 7. Economic System Justification | .77 | 4.29 | 1.09 | .01 | .17*** | -.07** | .17*** | .11*** | -.11*** | – |  |  |  |
| 8. Social Dominance Orientation | .85 | 2.51 | 1.24 | .00 | .15*** | .01 | .26*** | .16*** | -.07** | .70*** | – |  |  |
| 9. Support for Econ. Inequality | .84 | 2.69 | 1.34 | .00 | .06* | -.14*** | .11*** | .03 | -.11*** | .71*** | .70*** | – |  |
| 10. Ingroup Identification | .88 | 5.86 | 1.23 | .00 | .13*** | .09*** | .05* | .06* | .05* | .11*** | .00 | -.01 | – |
| 11. Trait competitiveness | .84 | 3.30 | 0.92 | .02 | .22*** | .15*** | .13*** | .14*** | -.06* | .26*** | .21*** | .13*** | .18*** |

*Notes*: Econ. = Economic; ****p* < .001, ***p* < .01, **p* < .05, †*p* < .10

Table S9

*Study 2: Standardized coefficient estimates of the simple slopes for the condition x moderator interactions on race-based psychological outcomes*

|  |  | High Moderator (+1 SD) | | Low Moderator (-1 SD) | |
| --- | --- | --- | --- | --- | --- |
| Moderator | Outcome | *β* | CI | *β* | CI |
| SDO | Perceived Interracial Competition | .29*** | [.22, .35] | .42*** | [.36, .48] |
|  | Perceived Discrimination | .13*** | [.06, .19] | .27*** | [.21, .34] |
|  | *Perceived Behavioral Avoidance* | .11** | [.04, .17] | .19*** | [.12, .25] |
|  | Perceived Interracial Mistrust | .06† | [-.01, .12] | .16*** | [.10, .23] |
| SEI | Perceived Interracial Competition | .28*** | [.22, .34] | .43*** | [.37, .49] |
|  | Perceived Discrimination | .12*** | [.06, .19] | .28*** | [.22, .35] |
|  | *Perceived Behavioral Avoidance* | .11** | [.04, .18] | .19*** | [.13, .26] |
|  | *Perceived Intergroup* *Anxiety* | .16*** | [.09, .23] | .24*** | [.18, .31] |
|  | *Perceived Interracial Mistrust* | .07* | [.001, .13] | .16*** | [.09, .22] |
| ESJ | Perceived Interracial Competition | .31*** | [.24, .37] | .40*** | [.34, .46] |
|  | Perceived Discrimination | .13*** | [.06, .19] | .27*** | [.21, .34] |
| TCOMP | Perceived Discrimination | .14*** | [.07, .20] | .25*** | [.19, .32] |
|  | *Perceived Behavioral Avoidance* | .10** | [.03, .17] | .19*** | [.13, .26] |
|  | Perceived Interracial Mistrust | .06† | [-.001, .13] | .16*** | [.10, .23] |
| RIGap | Perceived Interracial Competition | .44*** | [.37, .50] | .30*** | [.23, .36] |
|  | *Perceived Discrimination* | .25*** | [.18, .31] | .16*** | [.09, .22] |
|  | Perceived Behavioral Avoidance | .22*** | [.15, .29] | .09** | [.02, .16] |
|  | Perceived Intergroup Anxiety | .26*** | [.19, .33] | .16*** | [.09, .22] |

*Notes*: SDO = Social Dominance Orientation, SEI = Support for Economic Inequality, ESJ = Economic System Justification, TCOMP = Trait competitiveness, RIGap = Racial Income Gap, ****p* < .001, ***p* < .01, **p* < .05, †*p* < .10; italicized outcomes are qualified by marginally significant interactions.
