# Supplementary material for: The effect of perceived interracial competition on psychological outcomes
Source: PLoS One. 2021 Jan 29;16(1):e0245671. doi: 10.1371/journal.pone.0245671 (PMC7845962; doi:10.1371/journal.pone.0245671)
Supplement: S2 File — (PDF) [file pone.0245671.s003.pdf]

ASPREDICTED

Created: 10/25/2018 02:31 PM (PT)
Public: 07/01/2019 08:31 AM (PT)

1) Have any data been collected for this study already?
No, no data have been collected for this study yet.

2) What's the main question being asked or hypothesis being tested in this study?
Perceptions of racial competition in one's ZIP-code causally influences perceptions of discrimination, behavioral avoidance, intergroup anxiety, and interracial mistrust for both Whites and Blacks. Specifically, those in the high perceived racial competition group will report greater perceptions of discrimination, behavioral avoidance, intergroup anxiety, and lower interracial trust.
Regardless of condition, Blacks will report greater perceptions of discrimination, behavioral avoidance, intergroup anxiety, and lower interracial trust compared to Whites.

3) Describe the key dependent variable(s) specifying how they will be measured.
Perceptions of Racial competition adapted from 5-item Perceived competition scale (Murayama & Elliot, 2012).
Perceptions of Discrimination adapted from the 9-item Everyday Discrimination Scale (Clark, Coleman, & Novak, 2004).
Perceptions of Behavioral Avoidance adapted from 11-item Avoidance scale (Lackey, 2012).
Perceptions of Intergroup Anxiety adapted from 4-item state affect measure (Amodio, 2009).
Perceptions of Interracial Trust adapted from General Trust Scale (4-items; Yamagishi & Yamagishi, 1994).

4) How many and which conditions will participants be assigned to?
2 Conditions (High Perceived Racial Competition and Low Perceived Racial Competition)

5) Specify exactly which analyses you will conduct to examine the main question/hypothesis.
Independent Samples t-test will be conducted comparing the two conditions. Additionally, a 2x2 (Condition by Race) ANOVA will be conducted to examine a potential interaction effect. We are primarily interested in the main effects of condition and main effects of race. As for the interaction between race and condition, we have no a priori prediction, but are interested in analyzing.

6) Describe exactly how outliers will be defined and handled, and your precise rule(s) for excluding observations.
Participants that fail the attention check in the survey will be omitted from analyses. Moreover, participants that finish the survey is a very short amount of time (i.e. under 2 minutes) will be excluded. No analyses will be conducted with these individuals, and thus will be filtered a priori.

7) How many observations will be collected or what will determine sample size? No need to justify decision, but be precise about exactly how the number will be determined.
Using GPower 3.1.9.2, a t-test using the statistical test "Means: Difference between two independent means (two groups), with an estimated Cohen's d of .02, alpha of .05, and power of .8 yields a sample size of 394 per group. We will increase this number by 15% to account for anticipated participant exclusions due to failed attention checks. In addition, we will double it for 2 conditions, and double it again to have equal numbers of Whites and Blacks. Thus, the final N will be (394*1.15) x 2 x 2 = 906 x 2 = 1812.

8) Anything else you would like to pre-register? (e.g., secondary analyses, variables collected for exploratory purposes, unusual analyses planned?)
We will also measure 6 potential moderators of these effects, as these variables have been shown to be related to and meaningful with respect to the model. These variables are 1) ZIP-code level racial income gap, 2) Economic System Justification, 3) Group Identification, 4) Social Dominance Orientation, 5) Support for Economic Inequality and 6) Trait Competitiveness. We do not have any a priori hypotheses regarding specific direction, but believe that these variables may influence the impact of condition on our DVs.

Wharton University of Pennsylvania CREDIBILITY LAB
