# Supplementary material for: The effect of perceived interracial competition on psychological outcomes
Source: PLoS One. 2021 Jan 29;16(1):e0245671. doi: 10.1371/journal.pone.0245671 (PMC7845962; doi:10.1371/journal.pone.0245671)
Supplement: Attachment — Submitted filename: PONE-D-20-22299_reviewer.docx [file pone.0245671.s009.docx]

**Review PONE-D-20-22299**

**The effect of perceived interracial competition on psychological outcomes**

In this paper the authors answered a call by the field to investigate the causal nature of intergroup (specifically interracial) competition on negative psychological outcomes, specifically perceived discrimination, perceived behavioral avoidance, perceived intergroup anxiety, and perceived interracial mistrust. In Study 1 they recruited people of all racial groups, randomly assigned them to read about the Black-White perceived interracial competition in their local area as either being high or low, and filled out a series of measures tapping into negative psychological outcomes. In a pre-registered Study 2 they specifically recruited only Black and White participants and repeated the interracial competition manipulation.

It’s rare to read a paper that claims that there is a dearth of research on a fairly obvious topic and upon reflection realize there actually isn’t much work that specifically centers competition in intergroup relations! I do believe there is a fair amount of work using minimal group paradigms that manipulates competition, but that work focuses on behavioral or attitudinal outcomes, not necessarily these cognitive mechanisms discussed here.

Along many dimensions I enjoyed reading this manuscript. There were some places where things could be clearer (and I outline them below) but on the whole I think this is an important topic of inquiry. My main issue with this paper (and one that can likely be addressed by a lengthier discussion or with another study) is that I felt like the movement on their four psychological variables can be understood as additional manipulation checks rather than showing a causal pathway from perceived competition to perceived discrimination, behavioral avoidance, intergroup anxiety, and interracial mistrust. To their credit, the authors discuss this in the general discussion section (point four) but I wasn’t completely convinced that there was a large difference between their manipulation check and their four main DVs. The correlations for all but the mistrust items were rather high as well. In terms of what this alternative understanding could have looked like from a participant’s perspective; if you tell me that two groups are competing in my neighborhood, it makes perfect sense that these groups also discriminate against one another, avoid one another, have anxiety about one another, and mistrust one another. I would believe that purely based on my assumptions about how competition works, not at all because perceiving such competition has led me and my beliefs about racial relations to shift.

This alternative hypothesis is easier to generate because the manipulation and the DVs are so similar. The authors told participants that the average response to the question “Blacks and Whites are competing with each other” was either “completely” or “not at all” (i.e. telling participants that Black and White people have terrible or great relations with one another) and then effectively measured perceived relations between the two groups. Here is where *not* asking about the participants’ beliefs is a hindrance to testing the actual hypothesis. The “perceived” wording/third-party nature of the questions makes social desirability a concern. This becomes more important for Study 1 because there are various racial groups present but the study focuses specifically on the Black-White divide. There isn’t any real discussion around what it means for an Asian or a Hispanic person to answer these questions. The fact that condition (and not race) is the primary driver of the effects is another reason why I feel the alternative hypothesis – that participants are not actually changing their beliefs but reporting beliefs in line with the information told to them by the researchers – might have some merit. The work that does exist on how White people respond to threat (and experiencing competition is usually threatening) is varied. Sometimes they aggressively respond (think shifting demographics and status threat) while other times they move towards appeasement. The equal movement for anxiety, discrimination, mistrust, and avoidance for Black *and* White people was very surprising from that perspective.

I am a bit wordier than I usually am in reviews regarding this point because I’m trying to be as clear as possible. A lengthier discussion regarding how we should interpret the findings would go a long way to deal with my concern, or to run another study in which they manipulate beliefs about competition and assess participants’ actual discrimination and avoidant behaviors and/or feelings of mistrust and anxiety.

**Other (small) points:**

1. I had a hard time knowing who the participants’ were based on the abstract and the introduction. The authors wrote that they extended Study 1 in Study 2 by recruiting Black and White participants, which makes an assumption that Study 1 had only Black or White subjects. That’s actually not true so more clarity in writing about the population pool would be helpful.
2. The authors discussed a variety of intergroup process models but don’t include social dominance theory although it explicitly centers competition. I make this point because the authors do include several SDT-centered studies as evidence for their claims.
3. The authors discuss negative intergroup outcomes in a general sense throughout the introduction, but it was difficult to understand if the authors believed that there are negative outcomes for all groups in conflict, differential ones for low power versus high power groups, etc. This is part of why I thought Study 1 had only White subjects because some of these findings are specific to White participants. For example, the authors wrote “perceived interracial competition is associated with lower levels of support for affirmative action programs, higher levels of racial bias and stereotyping, ingroup favoritism, outgroup derogation, and perceived intergroup discord”. However, I assume that Black people don’t reduce support for affirmative action programs when they perceive interracial competition.
4. I loved the inclusion of Bayes factor calculations!

Reviewed by Sa-kiera T. J. Hudson, PhD

Postdoctoral Fellow at Yale University

[Sa-kiera.hudson@yale.edu](mailto:Sa-kiera.hudson@yale.edu)

Blame any and all typos on COVID-19
