# Supplementary material for: The effect of perceived interracial competition on psychological outcomes
Source: PLoS One. 2021 Jan 29;16(1):e0245671. doi: 10.1371/journal.pone.0245671 (PMC7845962; doi:10.1371/journal.pone.0245671)
Supplement: Attachment — Submitted filename: PONE-D-20-22299_R1_reviewer.docx [file pone.0245671.s010.docx]

**Review PONE-D-20-22299_R1**

**The effect of perceived interracial competition on psychological outcomes**

Thanks to the authors for carefully reading the reviewers comments and responding. There is substantial re-working on the discussion section in response to our concerns. I am still not 100% convinced that this manipulation is actually manipulating intergroup competition instead of introducing demand characteristics, but the focus on perceived competition and perceived outcomes is still an important addition to the literature.

There are some small theoretical clarification questions I had reading the manuscript but none of these points are critical to address in a final version.

1. On page 4, the authors write: “In response to threats from outgroups, ingroup members exhibit motivation to quell the threats, which can take the form of ingroup favoritism (e.g. entitlement justifications; Jost & Banaji, 1994), outgroup derogation (e.g. discrimination; Pratto & Lemieux, 2001), and behavioral avoidance (Esses et al., 2005).” I was confused with the juxtaposition of behavioral avoidance next to ingroup favoritism and outgroup derogation, as usually the literature compares behaviors that are ingroup versus outgroup focused assuming they encompass the full range of behaviors. Thus, avoiding the outgroup could be due to ingroup favoritism (e.g., wanting to be near similar others) or due to outgroup derogation (e.g., seeing the outgroup as inferior and wanting the “riffraff” segregated”).
   a. Similarly, in the next paragraph the authors discuss “discrimination” as an example of outgroup derogation yet ingroup favoritism can also lead to discrimination that has nothing to do with demoting the regard of the outgroup.
   b. I encourage the authors to review the manuscript to ensure that the theories and concepts presented are internally consistent.
2. The authors judiciously use italics, and it becomes difficult to understand what exactly the formatting technique is highlighting.
3. As I mentioned in the last review, the authors write “perceived interracial competition is associated with lower levels of support for affirmative action programs, higher levels of racial bias and stereotyping, ingroup favoritism, outgroup derogation, and perceived intergroup discord” but it wasn’t clear if the authors are arguing that both Black and White individuals show this effect. One suggestion is to only include the effects that are agnostic to the race/status of the individual, which allows the reader to place the lack of racial interactions within the existing literature. Furthermore, it more concretely explains their hypothesis that race would not matter, which was surprising to me as a reader.
4. I found the discussion of the four outcome variables on pages 25-26 useful to include earlier, around page 6, as it explains why these four variables are important to study in the context of perceived racial competition.
5. I was confused regarding the information in the brackets in the results sections: e.g., “t(839) = 9.58, [.86, 1.31], p < .001, d = .66.” Are they 95% Cis? If so, of what?
6. On page 13 the authors write: “On the one hand, because interracial (and intergroup) competition involves participation and engagement from both ingroups and outgroups, Blacks and Whites may be similarly influenced by perceptions of interracial competition. On the other hand, because Blacks experience worse outcomes compared to Whites across numerous social, psychological, and economic indicators, it is possible that perceived interracial competition disproportionally impacts Black participants”. It is also the case that because White individuals have more to lose if they feel competition, they might be disproportionally affected by competitive threats. The authors’ argument starts to include the status/power differential but feels like one side is missing.
7. Reading the work on pages 14 and 15, I had no idea why we were all of a sudden reading about race-based income inequality until the very last sentence. If possible, think about integrating the overview in the introduction or better introduce the why of including race-based income inequality beyond “One notable dimension in which Blacks and Whites exhibit a large disparity is in income”.
   a. You can easily move some version of this sentence to the beginning so that the reader understands why you are discussing race-based income inequality: “If race-based income inequality has the potential to enhance competition by making group differences and resource differentials salient, outcomes that stem from competition, including but not limited to perceived discrimination, avoidance, anxiety, and mistrust, may be exacerbated.
8. Is it possible that you found “The effects of condition on these outcomes were stronger for those living in areas with larger racial income inequality” because larger racial income inequality in one’s area made the manipulation more believable? If not, it would be useful to have a sentence or two debunking this and any other alternative hypotheses.
9. The authors bounce back and forth between active and passive voice. Not sure if it was intentional. As someone that tries to write in active voice but always slips into the passive, I wanted to flag in case that was something the authors wanted to address.

Reviewed by Sa-kiera T. J. Hudson, PhD

Postdoctoral Fellow at Yale University

[Sa-kiera.hudson@yale.edu](mailto:Sa-kiera.hudson@yale.edu)

Blame any and all typos on COVID-19
